# Family caregiver quality of life in multiple sclerosis among Kuwaitis: a controlled study

**DOI:** 10.1186/1472-6963-8-206

**Published:** 2008-10-07

**Authors:** Asmahan F Alshubaili, Jude U Ohaeri, Abdel W Awadalla, Asser A Mabrouk

**Affiliations:** 1 Department of Neurology, Ibn Sina Hospital, P.O. Box 2547, Safat, 13115, Kuwait; 2 Department of Psychiatry, Psychological Medicine Hospital, Gamal Abdul Naser Road, P.O. Box 4081, Safat, 13041, Kuwait; 3 Department of Psychiatry, Faculty of Medicine, Kuwait University, P.O. Box 24923, Safat, 13110, Kuwait

## Abstract

**Background:**

Research interest in the quality of life (QOL) of persons with multiple sclerosis (MS) has been spurred by the need to broaden outcome measures. Far less of this interest has been directed at the family caregivers, who bear most of the burden of care. The objectives of the study were: First, to compare the subjective QOL of family caregivers of persons with relapsing remitting and progressive MS, with those of a matched general population sample and caregivers of diabetes and psychiatric patients. Second, to assess the relationship of QOL with caregiver attitudes to MS and patient's variables.

**Methods:**

Consecutive MS clinic attendees were assessed with the 26 – item WHOQOL Instrument, and for depression and disability. Similarly, caregivers independently rated their own QOL as well as their impression of patients' QOL and attitudes to patients' illness.

**Results:**

The 170 caregivers, mean age 35.7 years, had no significant diagnostic differences in QOL domain scores and attitudes to MS. Caregivers had significantly lower QOL than the general population control group for five out of six domains and the general facet (P < 0.01), but higher QOL than the patients. When the scores were corrected for patients' depression and disability, caregivers had similar QOL with the general population group for four domains. Using corrected scores, MS caregivers had lower scores than diabetic and psychiatric caregivers in the physical, psychological and social relations domains. Majority expressed negative attitudes to MS. Caregiver QOL was more affected by their fear of having MS than their feelings about the illness and caregiving role. Caregiver attitudes had mostly no significant impact on their proxy ratings of patients' QOL. The significant predictor of caregivers' overall QOL was their impression of patients' QOL.

**Conclusion:**

Caregivers need specific attention if they are less educated, unemployed, afraid of having MS and caring for patients with longer duration of illness and less education. In particular, attention to patients' depression and disability could improve caregivers' QOL. Caregivers need specific programs to address fear of having MS, negative attitudes to illness and their unmet needs.

## Background

Research interest in the quality of life (QOL) of persons with multiple sclerosis (MS) has been spurred by the need to broaden outcome measures to include factors that might indicate less obvious disease burdens [1,2]. Far less of this interest has been directed at the family caregivers [1,3]. It is important to study the impact of caregiving on families because MS places substantial burdens on patients and caregivers [4]. Families have to cope with the presence of the disease, the added fact of an unpredictable prognosis, and the possibility that the patient may become severely physically and cognitively impaired [5]. Since a quarter of patients require caregivers to perform activities of daily living, it is not surprising that patients' health-related QOL is inversely correlated with caregiver burden [6].

Our review of the literature showed that all the reports on caregiver QOL in MS have emanated from the temperate/mediterranean countries of Europe and North America, where the disease is traditionally thought to have a higher prevalence and severity[7], compared with countries in the relatively lower latitudes, such as the Arab world[8,9].

Kuwait, a city-state in the Persian Gulf, is one of the relatively low latitude countries where a rising incidence and prevalence of MS has been reported [8]. A study of QOL among Kuwaitis is valuable because it presents the perspectives from a country where (compared with countries in temperate/mediterranean climates) the disease seems to have an earlier age at onset and relatively milder clinical severity [8,9]. In addition, an effective tax-free national social welfare system is in place. Hence, all treatments are provided free-of-charge. In addition, family social support is much available in the conservative culture.

It would, therefore, be interesting to see whether all these favorable factors would make for relatively good QOL among caregivers of MS patients, in comparison with a socio-demographically matched general population sample.

Another deficiency in the literature is the paucity of information on the relationship of family caregiver's attitudes to the illness and the impact of caregiver's impression of the patient's QOL on the QOL of the caregiver [10,11]. This issue of caregiver impression of patient's QOL (called proxy rating) is important in MS because of the widely reported cognitive impairment among the patients [2,12], the consequent unawareness of functional deficit, and their impact on the well-being of patients and caregivers [13].

### Objectives

The objectives of the study were as follows:

- Using the 26-item WHO QOL Instrument (WHOQOL-Bref), to compare the QOL ratings of caregivers of persons with relapsing remitting (RRMS) and primary/secondary progressive MS (PMS), with those of a socio-demographically matched general population sample

- to assess the association of the following variables with the caregiver's QOL: patient and caregiver demographic factors, caregiver attitudes to MS, as well as patient's duration of illness, depression, and physical disability

- to assess the factors that predict caregiver subjective QOL

The results were compared with those of previous studies, from a similar culture, of caregivers of patients with diabetes mellitus [14] and psychiatric disorders [15], who were also assessed with the WHOQOL-Bref.

### Hypotheses

In line with the objectives, we hypothesized as follows: First, caregivers of patients with RRMS would have significantly higher QOL domain scores and more positive attitudes towards MS, compared with caregivers of patients with PMS. This is because of the presumed greater severity of burden of caring for PMS [4-6]. In view of the availability of national welfare and family supports, however, caregivers would have similar scores with the control group in the related QOL domains (i.e., social relations & environment domains of WHOQOL-Bref). Caregivers would evidence lower QOL than the control group in the domains related to physical and psychological distress (i.e., physical & psychological health domains), because of the impact of caregiving [6]. Second, patients' depression and disability scores would be significantly associated with caregivers' QOL domain scores [16-18]. Furthermore, caregivers who expressed negative attitudes towards MS would have significantly lower QOL. That is, attitudes to MS are significant covariates of QOL. Third, the most significant predictor of the caregivers' QOL would be the caregivers' impression of the patients' QOL [14,16,19].

The clinical relevance of these hypotheses is that they could help to define a subset of caregivers whom clinicians need to give focused attention. Furthermore, they could help clinicians to identify the characteristics of caregivers which psychosocial interventions should target to make for improved quality of care.

## Methods

### Operational definition

We accepted the WHO definition of QOL as individuals' perception of life in the context of the culture and value system in which they live and in relation to their goals, expectations, standards and concerns [20]. Our focus was on subjective QOL, as distinct from objective QOL [15].

### The setting

The study took place in 2005/2006, at the outpatient clinic of the Neurology Department, Ibn Sina Hospital, which is the national hospital for neurology and neurosurgery in Kuwait. This hospital provided the sample for the national epidemiologic study of MS [8,9].

### Subjects

The patients were consecutive outpatient clinic attendees who fulfilled the study's inclusion criteria. First, the patients had been formally diagnosed for at least six months, using the Poser Criteria [21]. Second, each patient was accompanied by at least one family member or friend who lived with them in daily contact, was responsible for caring for the patient at home and could complete the questionnaires in Arabic. The adult family member who lived in closest caregiving interaction with the patient was interviewed [2]. The caregivers consisted of spouses, parents, siblings, and members of the extended family (e.g., uncles/aunts). These family caregivers are not specifically paid for looking after the patients at home. They regard it as a family obligation towards their loved ones.

The general population control group was selected by quota sampling from our WHOQOL-Bref data base for Kuwait, to match the caregivers by sex, age, occupation, marital status and level of education. In addition, MS caregivers' QOL domain scores were compared with those of family caregivers of patients with diabetes mellitus and psychiatric disorders who were assessed in a similar manner [14,15].

### The WHOQOL – Bref

This is a 26 – item self – administered generic questionnaire, a short version of the WHOQOL – 100 scale [20]. It can be analyzed from the perspective of either six domains (physical health, psychological health, level of independence, social relationships, environment, & spiritual) or four domains (physical health, psychological health, social relations, & environment). We used both models. The items on "overall rating of QOL" (OQOL) and "subjective satisfaction with health" are not included in the domains, but are used to constitute the "general facet on health and QOL" (general facet).

### Modification of the WHOQOL – Bref for the impression of caregivers

In order to produce the version of the WHOQOL – Bref with which the family caregivers rated their impression of the patients' QOL, we used the method of Sainfort et al [22], by giving a new direction to each item. By this modification, the caregiver could rate the patient as an observer. The modification of the WHOQOL – Bref was thus minimal[14,15,22].

The internal consistency of the WHOQOL – Bref, as assessed by Cronbach's alpha coefficient for the responses of all caregivers was high (0.89 for the original WHOQOL-Bref, and 0.93 for the caregiver impression version). In the Arab setting, the WHOQOL-Bref has been shown to have highly significant validity indices [19].

### Other assessments

The patients were also assessed with the 21 – item Beck's Depression Inventory (BDI) [23] and the expanded disability status scale [24]. On the basis of clinical experience with the caregivers and research experience in this field [8,25], we assessed their attitudes to the patients' illness by seeking their responses to the following four items: (i) caregiver feeling sad about the patient's illness; (ii) caregiver feeling disgusted about patient's illness; (iii) caregiver feeling tired and exhausted about caring for the patient; and (iv) caregiver feeling anxious about the possibility of having MS. The response options were: not present; a little; moderately; a lot. We took the following steps to articulate the caregiver attitudes' questionnaire. First, we examined the content validity by presenting the draft to doctors and nurses in neurology for their comments on the appropriateness and phrasing of the items. Thereafter, literate family caregivers and patients (not part of the main study) were requested to comment on it. All these comments were used to fine-tune the questions and produce the final document.

The internal consistency of the caregiver attitudes' questionnaire and BDI, as assessed by Cronbach's alpha coefficient for the responses of all subjects was significant (0.79 for the caregivers' attitudes, and 0.93 for the BDI).

### Data collection procedure

The questionnaires were translated into Arabic by the method of back – translation and have been used in recent studies in Arab settings to assess chronically ill patients and their family caregivers [14-19]. In a pilot exercise, the instruments were found to be suitable to the cultural setting.

The completion of the WHOQOL-Bref, the BDI and attitudes of caregivers' questionnaires by the subjects was supervised by a trained female Arab research assistant. One neurologist made all the EDSS assessments. At the preliminary stage of the study, the research assistant was trained in the use of the questionnaires using patients who did not participate in the main study. The study commenced when the research team was satisfied that the research assistant could confidently administer the questionnaires to patients. Patients and caregivers completed the questionnaires privately and without interference from the research assistant, after clarification of the objectives of the study and the meaning of the items. Illiterate patients were assisted by their educated relatives to complete the questionnaire, after the caregiver had completed his or her own. The few illiterate relatives were assisted by the research assistant who read out the questions and rated the responses, as recommended by the WHOQOL group [20]. Literacy in Arabic language is very high among Kuwaiti nationals.

Ethical approval for the work was obtained from the Faculty of Medicine, Kuwait University, and Ibn Sina Hospital, Kuwait. Patients and family caregivers gave verbal informed consent after the objectives of the study had been explained to them. They were duly informed that there would be no negative consequences for declining to participate. As is well known in our culture for such non-invasive studies[14,15], all families approached freely consented to participate in the study, especially as the approach was made by clinic staff in charge of the cases.

The physician in-charge of each case assisted the research assistant to obtain the relevant clinical data.

### Data analysis

Data were analyzed by the SPSS – version 11 (SPSS Inc., Chicago, Illinois). For the first hypothesis, summary scores were generated by organizing the items of the WHOQOL-Bref into the six domains and four domains previously highlighted. We compared mean differences in domain scores for caregivers of RRMS and PMS patients by independent sample t-test and standardized effect size calculations (noting 95% confidence intervals). QOL domain scores of the caregivers (as a group) were compared with those of the matched general population group using independent sample t-test and effect size calculations. In addition, we used chi-square tests to assess the association between caregiver attitudes, caregiver gender and patient's diagnosis. For the second hypothesis, the relationship between age (caregivers' and patients'), patients' duration of illness, depression score, EDSS score and caregiver QOL domain scores was assessed by Pearson's correlation. The association between other socio-demographic variables (level of education, occupation and marital status) and QOL was assessed by one-way ANOVA. In view of the fact that a number of the socio-demographic and clinical variables were significantly associated with QOL domain scores in these uni-variable analyses, analysis of covariance (ANCOVA) was used to assess how these variables were associated with QOL domain scores in multivariate relationships. Thereafter, we used caregiver QOL domain mean scores, corrected for patients' depression and disability scores, to compare with the general population control group, once again.

We assessed the relationship between caregiver QOL and caregiver attitudes to MS in the following ways. First, t-test was used to assess the differences in QOL domain scores between caregivers who expressed positive attitudes (i.e., response option, "not at all/a little") and those who expressed negative attitudes (i.e., response option, "moderately/a lot"). Second, caregiver attitudes were entered as covariates in general linear model, with QOL domain scores as dependent variables. For the third hypothesis, the predictors of caregivers' QOL (based on caregivers' general facet on health & QOL as dependent variable) were assessed in step-wise regression analysis.

In order to compare the present results with those of caregivers of diabetes mellitus patients [14] and psychiatric patients [15], we corrected the raw QOL domain scores (all the data are available to us) for age, sex, education, occupation and marital status of respective patients and caregivers, and patients' duration of illness. Thereafter, we compared the corrected means using one-way ANOVA and effect size calculations.

Missing data were handled by excluding cases analysis by analysis. All tests were two-tailed. A Bonferroni correction (P = 0.01) was used for multiple tests for univariate analyses; otherwise, the level of statistical significance was set at P < 0.05.

## Results

### Socio-demographic characteristics and attitudes to MS

The data for the MS patients have been presented elsewhere [17,18].

Over a period of seven months, 170 consecutive patient-caregiver dyad attendees at the clinic met our inclusion criteria and agreed to participate in the study. The caregivers consisted of 60 men and 86 women (the gender of 24 caregivers was not recorded), with mean age of 35.7 years. Parents and spouses constituted about a quarter, each, of the sample. There were no significant sex and diagnostic differences in age, education, and marital status (P > 0.05). Men were significantly more in formal employment than the women(P < 0.001). The caregivers were well matched with the general population control group by sex, age, education, occupation and marital status (P > 0.05) (Table 1).

**Table 1:** Comparison of socio-demographic characteristics: MS family caregivers vs. general population control

Socio-demographic Characteristics	All caregivers N = 170	General population control group N = 136 Men = 58 or 42.6%; Women = 78(57.4%)	X^2^	P
Age of caregiver (SD)	35.7(10.8)	35.7(10.5)		ns
≤ High school (%)	90(52.9)	72(52.9)		
≥ College (%)	80(47.1)	64(47.1)		ns
Unemployed/housewife (%)	54(38.0% of 142)*	55(40.4)		
Employed (%)	88(40.8% of 142)	81(59.6)	0.08	ns
Married (%)	104(71.2% of 146)	90(66.2)	0.6	ns

* N < 170 because of missing data.

Analysis of caregiver attitudes for the response options "moderate/a lot", showed that approximately two-thirds of caregivers expressed sadness about the patients' illness, while over one-half expressed feelings of disgust, exhaustion and fear of having MS.

There were no significant sex and diagnostic differences in caregiver attitudes to MS (P > 0.05).

### Differences in QOL domain scores

The men and women had similar QOL domain scores (P > 0.05). In most domains, the caregivers of patients with RRMS tended to have higher QOL domain scores than the caregivers of patients with PMS. But this trend did not reach significance (P > 0.05).

Using raw scores, the general population control group had significantly higher scores than the caregivers in all the domains (P mostly < 0.01), except the environment (P = 0.2) (Table 2). Similarly, caregivers had significantly higher scores than the patients for all the domains (P mostly < 0.001), except the environment (P > 0.05).

**Table 2:** Comparison of QOL domain scores of caregivers and general population control group

QOL domains WHOQOL-Bref	All caregivers: Mean(SD) N = 136–170	Control group: Mean (SD) N = 136	T	Df	P	Standardized effect size	95% Confidence interval
Physical health 6-domain model	10.5 (1.9)	11.1(2.0)	2.4	281	0.02	2.4	0.05 – 0.52
Psychological health 6-domain	16.9 (2.5)	17.7(2.9)	2.6	278	0.01	0.31	0.07 – 0.54
Independence	14.2 (2.2)	15.7(2.7)	5.3	281	0.001	0.63	0.38 – 0.86
Social relations	10.5 (1.8)	11.4(2.2)	3.9	279	0.001	0.46	0.22 – 0.70
Environment	28.2 (3.2)	28.9(4.5)	1.4	279	0.2	0.17	- 0.06 – 0.41
Spiritual	3.6 (0.8)	3.9(0.8)	2.9	281	0.004	0.34	0.11 – 0.58
General facet health & QOL	7.5 (1.0)	8.3(1.4)	5.4	283	0.001	0.64	0.4 – 0.88
Physical health 4-domain model	24.7 (3.8)	26.8(4.4)	4.2	280	0.001	0.51	0.27 – 0.75
Psychological health 4-domain	20.5 (2.9)	21.6(3.4)	2.9	276	0.004	0.35	0.11 – 0.59

### Comparison of MS caregivers' QOL with diabetes and psychiatric caregivers' QOL

Using means corrected for socio-demographic variables and duration of illness, we found that our MS caregivers had similar scores with caregivers of diabetic and psychiatric patients in the environment domain and general facet on health and QOL (P > 0.05). However, caregivers of psychiatric patients had higher scores than MS caregivers in the following domains: physical health (ES: 95% C.I. = 0.91: 0.67 – 1.16), and social relations (ES: 95% C.I. = 0.80: 0.55–1.04) (F = 15.3, df = 2/556, P < 0.0001). Caregivers of diabetes patients had higher scores than MS caregivers for the following domains: physical health (ES: 95% C.I. = 0.38: 0.12 – 0.63) and psychological health (ES: 95% C.I. = 1.08: 0.81 – 1.34) (F = 28.6, df = 2/556, P < 0.0001).

### Factors associated with QOL

The only noteworthy trend for the relationship between caregiver attitudes and QOL scores was that caregivers who felt afraid of having MS (moderately/a lot) had significantly lower scores in the physical health domain than caregivers for whom this fear was "not present/or a little" (t = 2.4, df = 113, P = 0.02; Effect size = 0.46, 95% C.I., 0.08 – 0.83).

For caregiver relationship to the patient, parents had significantly higher psychological health scores than spouses (F = 4.5, df = 4/104, P = 0.002).

Although caregiver QOL scores were negatively correlated with patient's BDI and EDSS scores and duration of illness, the level of significance did not meet our Bonferroni correction criterion for any of the domains (except: duration of illness versus spiritual domain: r = - 0.23, P = 0.01). In view of these trends, we did an initial ANCOVA in which the following were entered as covariates: socio-demographic characteristics of patients and caregivers, caregiver attitudes to MS, and patients' clinical data. Caregiver QOL domain scores were each entered as dependent variables. Of the many trends (Table 3A), the only significant covariates were as follows: caregiver's fear of having MS, lower levels of caregiver education (P < 0.008), caregiver unemployed, patient's longer duration of illness, and lower levels of education of patient (P < 0.05), all of which were associated with diminished caregiver QOL.

**Table 3:** Factors associated with caregiver quality of life domain scores in analysis of covariance (ANCOVA)

A. Significant covariates*	Carer QOL domains significantly associated in ANCOVA	F	P
Carer afraid of having MS	Physical health	8.6	0.008
Education of caregiver	General facet health & QOL	8.6	0.008
Occupation of caregiver	General facet health & QOL	4.6	0.04
Patient's duration of illness	Physical health	6.0	0.02
	General facet health & QOL	3.7	0.07
Gender of patient	Physical health	3.4	0.08
Education of patient	Environment	5.9	0.02
Occupation of patient	Environment	3.9	0.06
B. Significant covariates**			
BDI (depression) score	Social relations	4.0	0.05
	Environment	3.9	0.05
EDSS (disability score)	Independence	5.9	0.02

* Variables entered in ANCOVA: Carer's & pt's socio-demographic characteristics; carer's attitudes to MS; Pt's clinical data

** Variables entered in ANCOVA: Patient's depression & disability scores; duration of illness

With regard to caregiver attitudes, this result implies that caregivers were affected more by their concern about developing MS in the future, than their feelings about the patients' illness and their caregiving role.

When the ANCOVA analysis was repeated using only patients' duration of illness, BDI and EDSS scores as covariates (Table 3B), we found that these variables had significant impact on social relations, environment (for BDI) and independence domains(for EDSS) (P < 0.05).

However, the resulting corrected means from this later ANCOVA analysis are highly interesting. First, Table 4 shows that, after controlling for the impact of patients' BDI and EDSS scores on caregivers' QOL scores, the resulting corrected caregivers' domain scores were now lesser than those of the control group for only the following: social relations, spiritual and general facet (P < 0.001, ES: 0.35 – 0.62). Second, caregivers of patients with PMS had significantly higher physical health domain scores than the general population control group (P < 0.03). Third, caregivers of patients with PMS had significantly higher scores than caregivers of patients with RRMS for physical health and independence (P < 0.01).

**Table 4:** Adjusted caregiver QOL domain scores compared with general population control group*

WHOQOL-Bref domain	Corrected caregiver mean (SD) N = 120	General population Mean(SD) N = 136	T	Df	P	Effect size	95% C.I.
Physical health	11.3 (3.6)	11.1 (2.0)	0.6	254	0.5	0.08	- 0.17 – 0.32
Psychological health	16.9 (4.9)	17.7 (2.9)	1.5	254	0.1	0.19	- 0.06 – 0.44
Independence	15.3 (4.3)	15.7 (2.7)	0.9	254	0.3	0.12	- 0.13 – 0.36
Social relations	10.4 (3.8)	11.4 (2.2)	2.5	254	0.01	0.62	0.37 – 0.87
Environment	28.6 (6.2)	28.9 (4.5)	0.4	254	0.7	-	-
Spiritual	3.4 (1.4)	3.9 (0.8)	3.5	254	0.007	0.43	0.18 – 0.68
General facet health & QOL	7.7 (2.1)	8.3 (1.4)	2.8	254	0.005	0.35	0.11 – 0.60
Physical health:							
RRMS (N = 103)	10.5 (2.1)**	11.1 (2.0)****	2.2	151	0.03	0.51	0.00 – 1.01
PMS (N = 17)	12.2 (2.9)						
Independence:							
RRMS (N = 103)	14.2 (2.4)***	15.7 (2.7)****	0.9	151	0.3	0.25	-0.26–0.75
PMS (N = 17)	16.4 (3.4)						

* Caregiver's QOL domain scores were corrected for patient's depression (BDI) and disability (EDSS) scores

** Comparison: RRMS Vs PMS physical health: T = 2.9, df = 118, P = 0.004

*** Comparison: RRMS Vs PMS independence: T = 3.3, df = 118, P = 0.001

**** Comparison: PMS Vs general population control group

In other words, caregivers seemed to be at risk for lower QOL, if they were afraid of having MS, and were less educated, unemployed, and caring for patients with longer duration of illness, less education and significant depression and disability (Tables 3 &4). In addition, whatever negative attitudes they might have had towards MS, these attitudes had no significant impact on their QOL as a group; rather, their concern was a more personal one, namely, whether they would be stricken with the same disability and distress of MS that their relatives were suffering from.

### Predictors of QOL

In multiple (stepwise) regression analysis, with the caregiver general facet as the dependent variable and patient's and caregiver's characteristics as independent variables, the only significant predictor of the caregiver's QOL was the general facet derived from the caregiver's impression of the patient's QOL. This accounted for 10.6% of the variance (standardized beta = 0.33, P < 0.001).

In view of this result, it was necessary to assess the impact of caregiver attitudes to MS on the caregiver impression of the patient's QOL. This was done by a series of t-tests (with Bonferroni correction), using domain scores derived from caregiver impression of the patient's QOL as the dependent variables. Each caregiver attitude (positive versus negative) was used as a grouping variable. The only noteworthy result was that caregivers who felt sad about the patients' illness rated the patients as having significantly lower physical health scores than caregivers who did not express sadness about the patients' illness (t = 2.56, df = 106, P = 0.01; Effect size = 0.52, 95% C.I. = 0.11 – 0.92). It is important to note that caregiver attitudes had no significant impact on their impression of patients' general facet on health and QOL.

## Discussion

### Limitations and strengths of the study

The limitations of the study are that it was cross-sectional, involving only subjects from one center, and so the subjects may not be representative of the general population of caregivers of MS patients in Kuwait. In addition, we did not assess the patients' perception of the quality of care given in the family. However, the socio-demographic and clinical characteristics of our patients were much similar to those of the Kuwait epidemiologic sample [8], and the focus of this study was not on patients' satisfaction with services.

The other strengths of the study are that we were able to compare the MS caregivers with a gender-, age-, education-, occupation-, and marital status- matched general population group, and assessed the relationship of caregiver impression of the patient's QOL and caregiver attitudes to the patient, with the QOL of the caregiver. This is a rare methodology in the MS QOL literature. Furthermore, we compared our MS caregiver QOL scores with those of caregivers of patients with other chronic illnesses, who were similarly assessed in previous studies in our region.

### Differences in QOL domain scores

In analyzing for the first hypothesis, we found that, using the uncorrected scores, there were no significant gender and diagnostic differences in QOL domain scores. In line with previous reports, however, the caregivers had significantly lower scores than the general population control group in most domains [5,6,13,26,27]. We had hypothesized that the combination of social supports and relatively milder disease severity pattern would be associated with relatively lighter caregiver burden and consequent caregiver QOL, similar to that of the general population in the social relations and environment domains. However, it has been shown that even for persons with relatively mild disease, QOL deteriorates early in the course of illness [28]. It has been suggested that the high prevalence of psychiatric morbidity (especially depression) among MS patients makes them and their caregivers vulnerable to diminished QOL[5,29,30]. Hence, studies comparing QOL across chronic medical illnesses have found that MS patients have lower QOL than other patient groups[31], a situation underscored by the relatively high risk of suicide among MS patients[32]. In line with this, our MS caregivers had significantly lower scores than Sudanese caregivers of diabetic and psychiatric patients in the domains of physical health, psychological health and social relations.

Using MS as a model, it has been shown that a chronic neurological disorder has the potential to produce psychosocial consequences at the initial stages of illness, because of its impact on self-esteem, civil status, social and leisure activities [33].

Another possible contributor to the lower QOL among caregivers is the impact of their attitudes to the illness and the emotional undercurrents at home. In a study employing the focus group approach, caregivers described the influence of MS on their own occupational status, their non-acceptance of the disease, a perception of lack of support by other members of the family, as well as what was considered to be selfish and intransigent attitudes of the patients[30]. While this finding may resonate with our data showing that majority of caregivers expressed negative attitudes towards the illness, our t-tests showed that the only caregiver attitude associated with diminished QOL was the fear of having MS in the future. This was supported by the ANCOVA data, which showed that, of the caregiver attitudes, the only significant covariate of QOL was caregiver fear of having MS. In other words, despite their expressed negative attitudes towards MS, the burden of caregiving did not significantly diminish their subjective satisfaction with most areas of living experience (i.e., QOL as assessed by the WHOQOL-Bref). This dissonance between the burden of caregiving and satisfaction with the caregiving role is well known in the literature [25].

As partial support for our first hypothesis, however, caregivers and the general population control group had similar scores in the environment domain. The constituent items for this domain include: money for needs, satisfaction with transport, safety, and information for health. The similarity of the scores of caregivers and control group in the environment domain could be taken as an indication that the national social welfare provisions did have some positive impact on this sample of caregivers.

### Factors associated with QOL

In analyzing for the second hypothesis, using ANCOVA, we replicated the evidence in the literature that, patients' and caregivers' characteristics mutually interact. In particular, MS patients' depression and disability have been shown to have adverse effects on the QOL of caregivers [5,29,30]. Furthermore, it appeared that the available national welfare supports were not sufficient to lift the QOL of caregivers who were less educated, unemployed, and caring for patients with longer duration of illness. Coupled with the finding of the impact of caregiver fear of having MS, our data support the call for clinicians to routinely pay specific attention to the needs of MS caregivers, and utilize the services of the social welfare department of the hospital for vulnerable groups[29].

### Predictors of QOL

In analyzing for the third hypothesis, we replicated previous findings about the predictive power of caregiver impression of patient's QOL on the QOL of the caregiver in chronic medical populations [14-16]. In view of the robustness of this finding, it was necessary to examine whether caregiver proxy rating of the patient's QOL was influenced by caregiver negative attitudes towards MS. This is because of the impression in the literature that the individual's perception of MS could be a major factor contributing to QOL [34]. Our finding that caregiver attitudes mostly had no significant impact on their rating of the patient's QOL, adds to the reliability of the predictive power of caregiver impression of the patient's QOL.

In order to explain this finding, we suggest that in the same way that family caregiver adverse emotional reactions have been found to predict relapse for severe psychiatric illnesses [35], caregiver positive appreciation of the patient's QOL could impact on the QOL of the patient and that of the caregiver. Furthermore, we suggest that recent brain -behavior findings about "mirror neurons"[36] and the phenomenon of "social intelligence" indicate that the patient- caregiver dyad interaction and its association with QOL has roots in the neurology of human behavior[36,37]. In other words, the caregiver's assessment of the patient mirrors not only the patient's QOL, but also is a window into how the burden of caring affects their own psychosocial living experience. Hence, in a study of "benefit finding" (defined as the identification of benefits in adversity) among carers of MS patients, it was shown that carer benefit finding was related to carer positive adjustment [38].

## Conclusion

Our data indicate that caregivers are vulnerable and need specific attention, if they are less educated, unemployed, afraid of having the illness, and caring for patients with longer duration of illness and less education. Attention to the treatment of depression, and rehabilitation for disability among the patients has the potential to improve caregiver QOL. The findings support the call for a specific psychosocial program for caregivers, to address negative attitudes to illness and their unmet needs, with a view to enhancing their caregiving role and QOL.

## Competing interests

The authors declare that they have no competing interests.

## Authors' contributions

AFA, JUO and AWA jointly designed the study, analyzed the data and wrote up the manuscript. AAM made the clinical assessments, including EDSS. JUO and AWA trained the research assistant. AFA and AAM supervised the interviews, and ensured correct diagnosis and other clinical data. All authors read and approved the manuscript.

## Pre-publication history

The pre-publication history for this paper can be accessed here:


